# Influence of biotin intervention on glycemic control and lipid profile in patients with type 2 diabetes mellitus: A systematic review and meta-analysis

**DOI:** 10.3389/fnut.2022.1046800

**Published:** 2022-10-31

**Authors:** Yujia Zhang, Yiwang Ding, Yawen Fan, Yenan Xu, Yuting Lu, Lingzi Zhai, Ling Wang

**Affiliations:** Faculty of Medicine, Macau University of Science and Technology, Macao, Macao SAR, China

**Keywords:** biotin, vitamin B, glycemic control, lipid profile, T2DM

## Abstract

**Background:**

Biotin is a water-soluble vitamin acting as a covalently bound coenzyme in regulating energy production. Previous studies have reported that biotin supplementation may influence blood glucose and lipid level in patients with type 2 diabetes mellitus (T2DM).

**Methods:**

We searched Pubmed, Embase, and Cochrane library databases up to 8th August 2022 for studies examining the effects of biotin supplementation in T2DM patients. Pooled effects were measured by weighted mean differences (WMDs) with 95% confidence intervals (CI) using random effects models. Inter-study heterogeneity was assessed and quantified.

**Results:**

A total of five random controlled trials (RCT), involving 445 participants were included. It was suggested that biotin supplementation for 28 to 90 days significantly decreased the level of fasting blood glucose (FBG) (MD: −1.21 mmol/L, 95% CI: −2.73 to 0.31), total cholesterol (TC) (MD: −0.22 mmol/L, 95% CI: −0.25 to −0.19) and triglycerides (TG) (MD: −0.59 mmol/L, 95% CI: −1.21 to 0.03). No significant beneficial effects were observed on insulin (MD: 1.88 pmol/L 95% CI: −13.44 to 17.21). Evidence for the impact of biotin supplementation on the levels of glycated hemoglobin (HbA1c), low-density lipoprotein cholesterol (LDL-C), high-density lipoprotein cholesterol (HDL-C) and very low-density lipoprotein cholesterol (VLDL-C) was limited to draw conclusion.

**Conclusions:**

Biotin supplementation may decrease FBG, TC and TG levels. However, its influence on insulin is not significant and further studies on the effects of biotin on HbA1c, LDL-C, HDL-C and VLDL-C are expected.

## Introduction

Diabetes mellitus is a global health problem with a prevalence of 11.3% and is projected to rise continuously (1). According to the World Health Organization, 422 million people worldwide are suffering from diabetes and 1.5 million deaths are directly attributed to diabetes every year. Type 2 diabetes mellitus (T2DM) is the most common type of diabetes and usually results in macro- and micro-vascular complications and comorbidities in the body, such as diabetic retinopathy (DR), diabetic foot, and diabetic kidney disease (DKD) (2). These chronic complications have caused severe adverse impact on the quality of life of human beings. Therefore, the prevention and management of T2DM is of vital importance to alleviate its disease burden.

Dietary vitamin supplementation is an easy and economic strategy for both pre-diabetic and diabetic patients to help control glucose and lipid metabolism. Vitamins are generally classified as fat-soluble and water-soluble. Various studies have systematically evaluated the effects of fat-soluble vitamins in lowering the risk of T2DM and improving glycemic indices, such as vitamin A (3), vitamin D (4–7), vitamin E (8) and vitamin K (9). Specifically, vitamin D supplementation has been proved to significantly ameliorate status of fasting blood glucose (FBG), homeostatic model assessment of insulin resistance (HOMA-IR), and glycated hemoglobin (HbA1c) (10). Similarly, the role of water-soluble vitamins is also important in maintaining energy metabolism. For example, vitamin B1 (thiamine) is a co-enzyme for several enzymes, including transketolase which is essential in the non-oxidative branch of the pentose phosphate pathway (PPP) (11). Many diabetic patients have been shown to have thiamine deficiency and lower blood thiamine pyrophosphate concentrations were associated with higher risk of DR (12). Moreover, vitamin B12 deficiency, usually caused by prolonged use of metformin, could result in severe oxidative stress and peripheral neuropathy (13, 14). As frequent urination and excessive thirst are two of the main symptoms of T2DM patients, it is unknown whether water soluble vitamins would be lost along with the extra urine discharge, thereby exacerbate the water-soluble vitamin deficiency in T2DM patients. Therefore, the study of individual water-soluble vitamin supplementation is necessary to determine their blood status and effects on T2DM patients.

Biotin, or vitamin B7, is a water-soluble vitamin that acts as a prosthetic group of carboxylases (15). It serves as the essential cofactor for the 6 biotin-dependent carboxylases: acetyl-CoA carboxylase (ACC), geranyl-CoA carboxylase (GCC), 3-methylcrotonyl-CoA carboxylase (MCC), pyruvate carboxylase (PC), propionyl-CoA carboxylase (PCC), and urea carboxylase (UC) (16). The first physiological function of biotin was found in 1968 and proved to increase hepatic glucokinase transcription (17). Subsequently, many *in vitro* studies reported that biotin could stimulate pancreatic islet glucokinase activity and expression (18), increase insulin secretion (19, 20), and induce insulin receptor synthesis (21). Meanwhile, various *in vivo* studies have also proven the efficiency of biotin in ameliorating diabetic status. Lazo et al. (22) investigated the effects of biotin in rodent pancreatic islets and confirmed that biotin supplementation could augment the proportion of beta cells and suppress mRNA expression of neural cell adhesion molecule. In animal studies, the results of glucose tolerance test (GTT) and insulin tolerance test (ITT) also supported the association between biotin treatment and improved tolerance condition (23, 24).

Current researches have demonstrated that biotin deficiency could impair energy production by decreasing glucose utilization and oxidative phosphorylation (25). Chuahan et al. (26) reported that biotin could regulate the glucokinase gene at the transcriptional stage in starved rats. In addition, insulin expression and secretion were found to be increased in response to biotin administration (18). On the other hand, excessive biotin intake may also ameliorate diabetic status. A study conducted in 43 Japanese T2DM patients demonstrated a decrease of approximately 45% of FBG concentration after one month of oral supplementation of 9 mg of biotin per day (15, 27). Similar effects were also observed in type 1 diabetic patients whose FBG levels decreased up to 50% after daily administration of 16 mg biotin for one week (28). High-dose biotin may compensate for subnormal insulin exposure by suppressing FOXO1 levels (29). Although the mechanism of hyperglycemia is different, biotin is effective in both type of diabetes mellitus (18, 30, 31). Moreover, many clinical trials have shown the hypoglycemic effect of biotin supplementation in overweight and obese individuals with T2DM (32, 33). A double-blind placebo-controlled trial including 348 participants reported a significant decrease in LDL-C, TC, HbA1c, and VLDL-C after 3 months intervention (34). However, another biotin intervention lasted for 4 weeks showed no significant change in plasma glucose, insulin, TG, TC or lactate concentration compared with placebos (35). The difference between these results may be caused by the small number of participants in one study and the specific conditions of the experiment such as the duration and dosage of the intervention.

Therefore, considering the lack of consensus, we performed a systematic review and meta-analysis for random controlled trials (RCTs) investigating the effects of biotin supplementation on glycemic control, including HbA1c, FBG, insulin, and lipid profile in T2DM patients. Prior to our research, no meta-analysis has been conducted in this regard.

## Methods

This systematic review and meta-analysis was performed based on the Preferred Reporting Items for Systematic Reviews and Meta-Analysis reporting guidelines (36).

### Literature search strategy

We searched the Pubmed, Embase, and Cochrane library electronic databases to identify RCTs that reported the effects of biotin on glycemic control in patients with type 2 diabetes, through 8th August 2022. The following medical subject headings (MeSH) terms and non-MeSH search terms were included: ((“Biotin” OR “Vitamin B7” OR Water-Soluble Vitamins) AND (“Type 2 diabetes” OR T2DM OR diabetes mellitus OR non-insulin dependent diabetes) AND [Intervention OR randomized OR trial OR “controlled trials” OR “clinical trials” OR “cross-over” OR parallel)]. The detailed search strategy is listed in the Supplemental materials. Articles satisfying the intervention, ending point, and study design criteria were pulled. We also manually reviewed the reference lists of the included studies to avoid missing related researches.

### Study selection

Two authors (ZY and FY) independently screened the titles and abstracts of every paper retrieved by the literature search to identify potentially eligible studies. We excluded studies that are letters, comments, conference papers, meta-analysis, reviews, RCTs with duplicate data or studies with insufficient data. RCTs that reported at least one of the following outcomes were included: 1) HbA1c, 2) FBG level, 3) insulin level, 4) TC level, 5) TG level, 6) HDL-C level, 7) LDL-C level, 8) VLDL-C level, 9) TG/HDL-C ratio. Any discrepancies regarding on the selection process were resolved by a third author (DY).

### Data extraction

Data extraction was conducted by two authors independently (ZY and DY). The following data were extracted from each study: first author, publication year, location of the study, study design and duration, age, gender, sample size, type of intervention, the doses of biotin supplementation, BMI, main glycemic control (mean and SD) for both control and supplementation groups at baseline and end of the studies. Outcomes that were measured in different units were unified by mathematical conversion.

### Quality assessment

Cochrane criteria was applied to assess the risk of bias and quality of the included studies (37). The assessment criteria include random sequence generation, allocation concealment, blinding of participants, blinding of outcome, incomplete outcome data, selective report and other bias. Each study was classified as low, high or unclear risk of bias regarding on each segment.

### Statistical analysis

All statistical analysis was performed using Review Manger V5.4 (Copenhagen: The Nordic Cochrane Center, Cochrane). To estimate the effect size of biotin on glycemic control, random-effects model was used to evaluate the WMD, SD and corresponding 95% confidence intervals (CI). Statistical heterogeneity was assessed in the meta-analysis using the *I*^2^ and χ^2^ statistics, and heterogeneity was considered substantial if *I*^2^ >50% and *P-*value of <0.10 in the χ^2^ test (38). A sensitivity analysis was performed by removing each study one by one, to explore the contribution of each study to the overall mean difference. Subgroup analysis was performed according to the dosage of intervention (<or ≥ 9 mg/d). Trials not applicable for meta-analysis were reported in a narrative form.

## Results

### Search results

**Figure 1:**
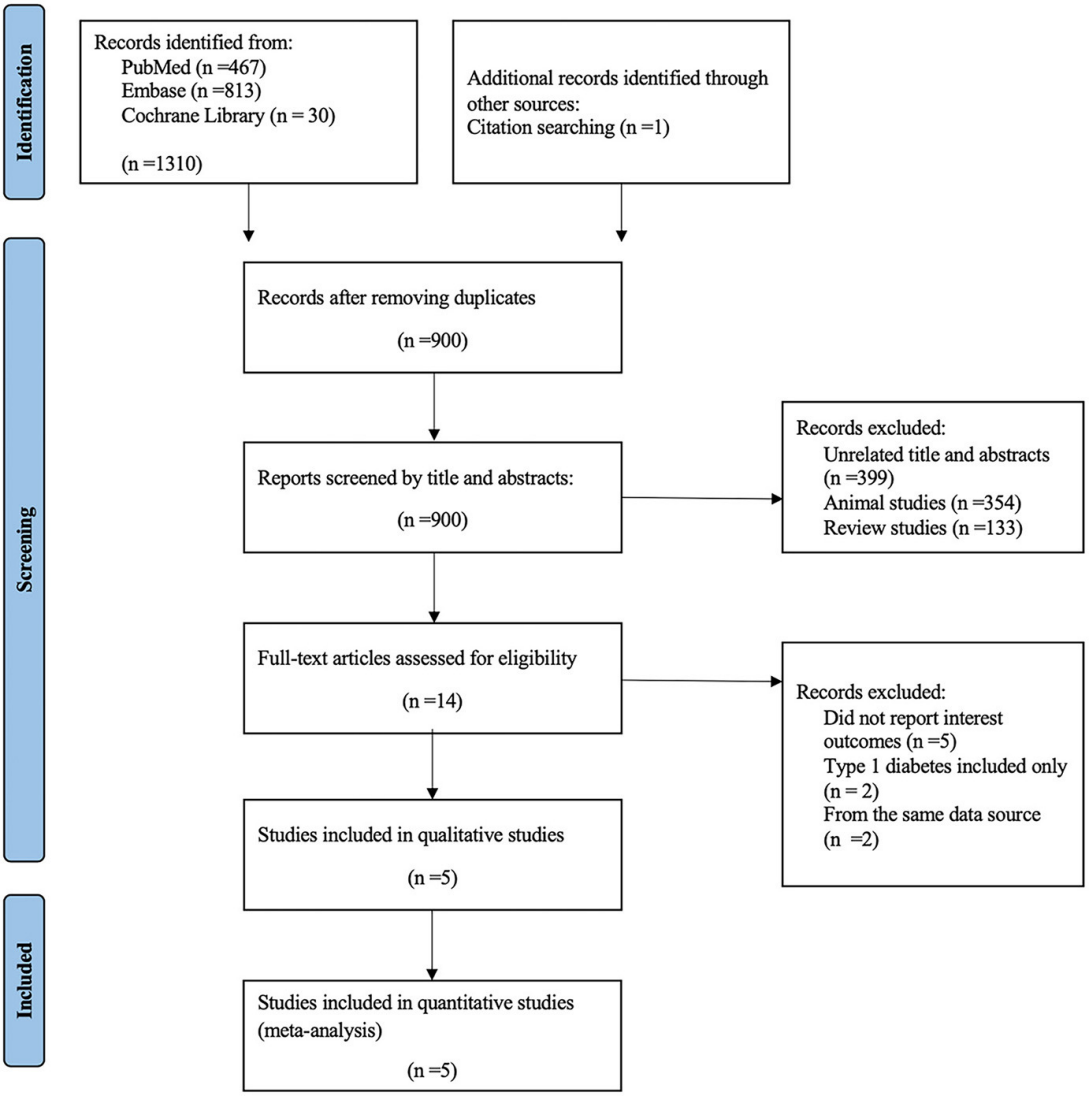
PRISMA flowchart of study selection.

A total of 1,310 articles were identified through database searching and citation searching, with 900 remained after removing duplicates. Among the remaining articles, 886 records were excluded due to the following reasons: unrelated title and abstracts, animal studies and review studies. After a full-text assessment for eligibility, five trials were included in the qualitative and quantitative analysis (Figure 1).

### Characteristics of the studies

The baseline characteristic of the included studies is demonstrated in Table 1. The five trials were conducted in three different countries including two in the United States, two in Mexico and one in Japan from 1993 to 2007. All of the trials were placebo controlled parallel RCTs and the intervention period varied from 4 weeks to 3 months, while 284 patients were in the experiment group and 161 were in the placebo group. One trial (27) did not report the gender of participants, while the male and female participants distribution was even in two trials (35, 39). But the other two trials (32, 34) showed male predominance and female predominance, respectively. Baseline mean age and BMI varied from 46 to 59 years and 28.6 to 30.5 kg/m^2^, respectively. The dosage of biotin intervention ranged from 1.5 mg/d to 15 mg/d.

**Table 1:** Baseline characteristic of the included studies.

**Authors**	**Year**	**Country**	**Design**	**Duration**	**Male (%)**	**Age (years)**		**BMI (kg/m** ^**2**^ **)**		**Intervention**		**Sample size**		**Reference**
						**EG**	**PG**	**EG**	**PG**	**EG**	**PG**	**EG**	**PG**	
Cristina et al.	2006	Mexico	Parallel	28 days	38.9%	48.6± 3.5	49.0 ± 3.7	29.7 ± 1.5	29.7 ± 1.4	15mg/d	Placebo	10	8	(32)
Cesar et al.	2007	United States	Parallel	90 days	59.7%	57.6 ± 10.1	59.6 ± 8.3	30.5 ± 3.3	30.4 ± 3.1	2mg/d biotin plus 0.6mg/d chromium	Placebo	226	122	(34)
Gregory et al.	2006	United States	Parallel	28 days	52.8%	53 ± 9	48 ± 9	30 ± 4	30 ± 4	2mg/d biotin plus 0.6mg/d chromium	Placebo	20	16	(39)
Armida	2004	Mexico	Parallel	28 days	48.7%	50.4 ± 7.7		28.6 ± 4.9		1.5mg/d	Placebo	10	5	(35)
Masaru	1993	Japan	Parallel	30 days	-	46 ± 10		-		9mg/d	Placebo	18	10	(27)

EG, experiment group, PG, placebo group.

### Quality assessment

**Figure 2:**
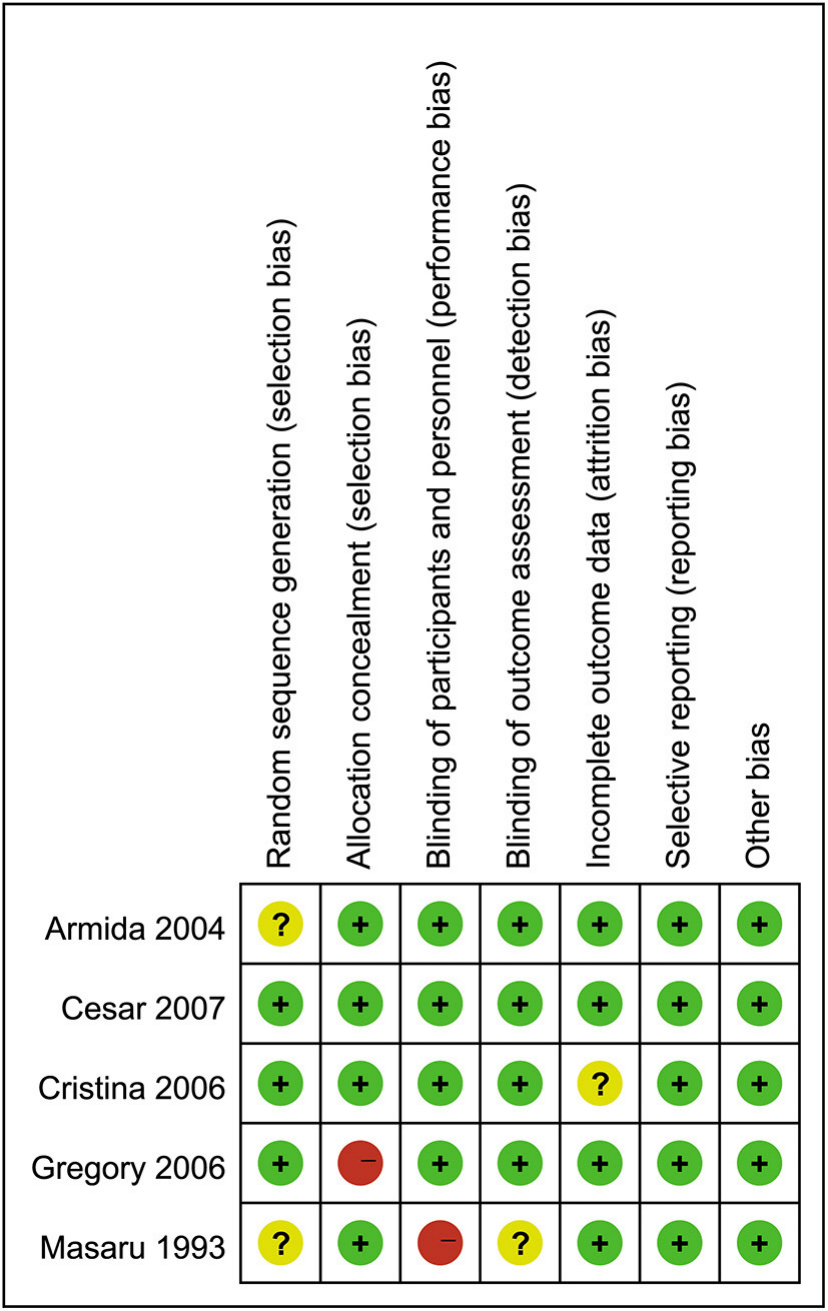
Quality assessment for the included studies.

The results of the quality assessment for the included studies are presented in Figure 2. Three trials had a rigorous experimental design and were considered to be at low risk. Two trials (27, 39) were considered to be at a moderate risk due to either unclear or lack of reporting on various potential biases.

The heterogeneity among studies for FBG (*I*^2^ = 0%, *P* = 0.33), insulin (*I*^2^ = 48%, *P* = 0.12), TC (*I*^2^ = 33%, *P* = 0.21), TG (*I*^2^ = 0%, *P* = 0.38), HDL-C (*I*^2^ = 0%, *P* = 1.00), LDL-C (*I*^2^ = 0%, *P* = 0.93) and TG/HDL ratio (*I*^2^ = 0%, *P* = 0.33) was not detected. Heterogeneity measured for VLDL-C (*I*^2^ = 88%, *P* = 0.004) was significant, which might be caused by using random effects model.

### Effects of biotin on FBG levels

**Figure 3:**
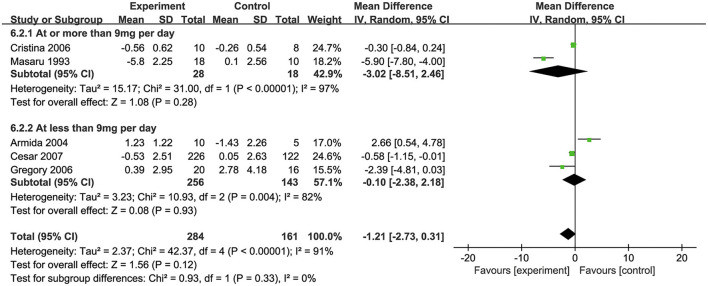
Effects of biotin supplementation on FBG levels <or ≥ 9 mg/d.

All of the five included studies examined the effect of biotin supplementation on FBG levels. Overall, biotin supplementation significantly reduced FBG levels (MD: −1.21 mmol/L, 95% CI: −2.73 to 0.31, *P* = 0.33, *I*^2^ = 0%). Regarding on subgroup analysis, two trials with supplementation of biotin dosage ≥ 9 mg/d significantly decreased FBG levels (MD: −3.02 mmol/L, 95% CI: −8.15 to 2.46). Three trials with supplementation dosage of <9 mg/d biotin demonstrated no significant difference compared with placebos (MD: −0.10 mmol/L, 95% CI: −2.38 to 2.18) (Figure 3). In addition, although two trials used the combination of biotin and chromium, subgroup analysis showed no significant difference in the effects of supplementation between the groups with or without chromium (34, 39) (Supplementary Figure S2).

### Effects of biotin on insulin levels

**Figure 4:**
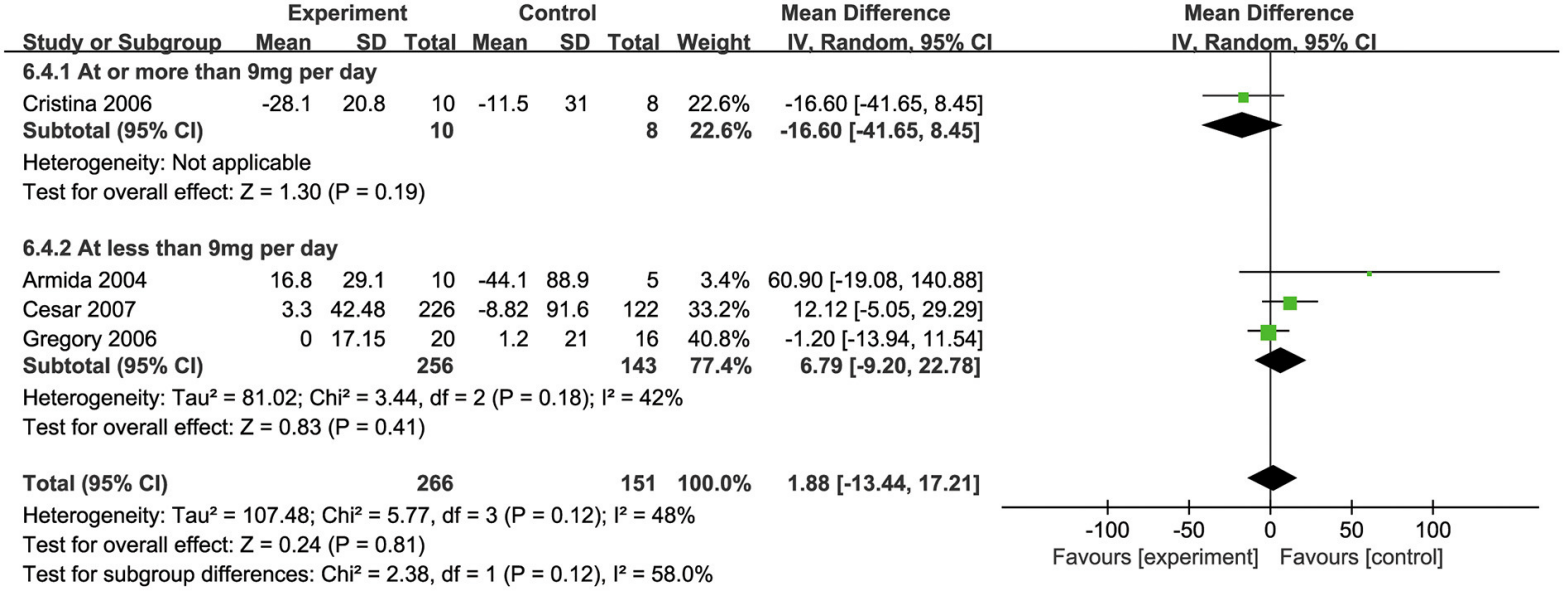
Effects of biotin supplementation on insulin levels <or ≥ 9 mg/d.

Four studies (32, 34, 35, 39) investigated the effects of biotin supplementation on insulin levels. Overall, no significant difference was observed in this meta-analysis (MD: 1.88 pmol/L, 95% CI: −13.44 to 17.21) (Figure 4). Regarding on subgroup analysis, one trial involved 18 participants, ten of which took biotin at the dosage of ≥ 9 mg/d and the other eight took the placebo, both groups showed a significant decrease in insulin levels, while the decrease was more remarkable in biotin group (MD: −16.6 pmol/L, 95% CI: −41.65 to 8.45). However, three trials with supplementation dosage <9mg/d biotin presented a contrary trend where insulin levels were raised after intervention (MD: 6.79 pmol/L, 95% CI: −9.20 to 22.78). Besides, the combination of biotin and chromium supplementation did not show any significant difference with the subgroup of individual biotin supplementation (34, 39) (Supplementary Figure S3).

### Effects of biotin on HbA1c levels

Only one trial (34) reported the effects of biotin supplementation on HbA1c levels in T2DM patients. Although it's not enough for a meta-analysis, according to the original article, the experiment group involving 226 patients showed a significant reduction in HbA1c levels compared with the placebo group (MD: −0.18%, 95% CI: −0.39 to 0.03).

### Effects of biotin on TC and TG levels

**Figure 5:**
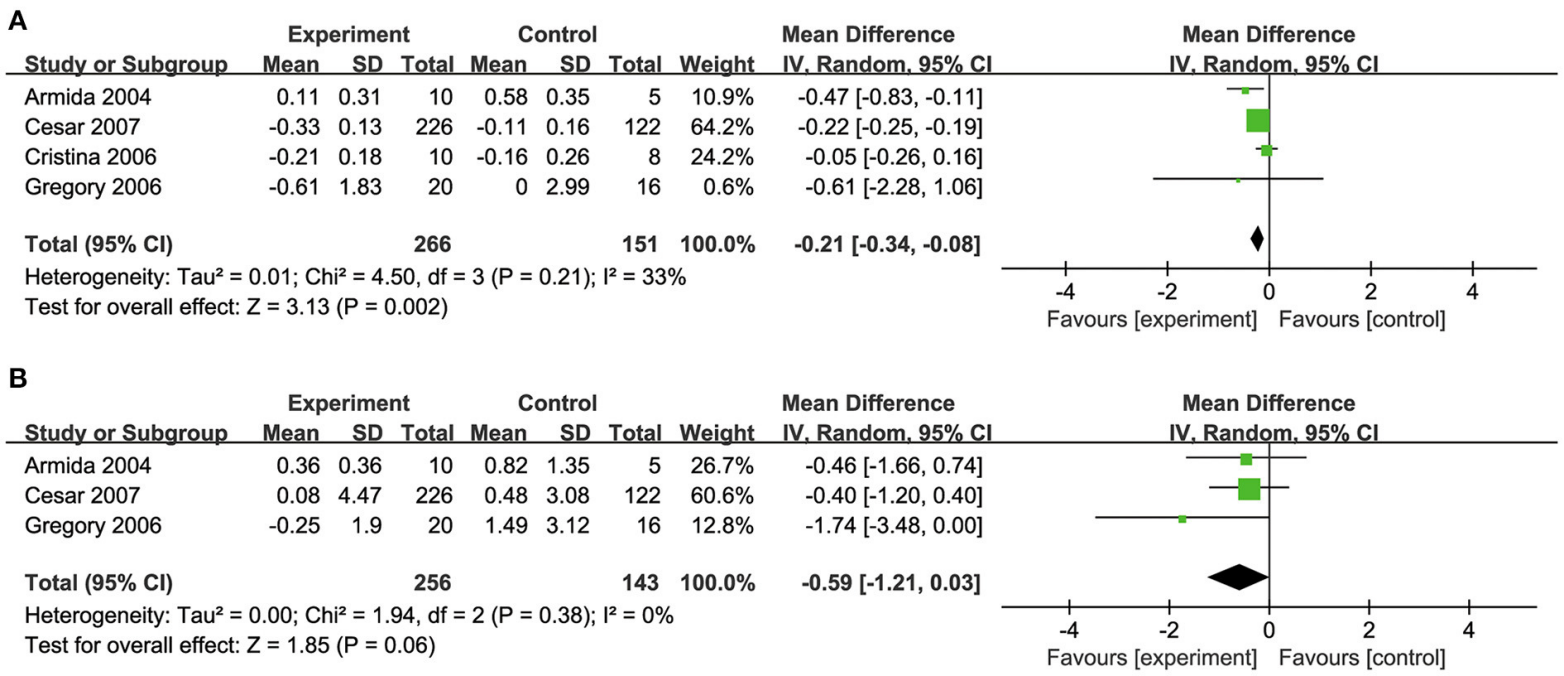
**(A)** Effects of biotin supplementation on TC levels. **(B)** Effects of biotin supplementation on TG.

Four studies (32, 34, 35, 39) investigated the effects of biotin supplementation on TC levels (Figure 5A) and three studies (34, 35, 39) reported TG levels (Figure 5B). Overall, biotin supplementation significantly reduced TC (MD: −0.22 mmol/L, 95% CI: −0.25 to 0.19) and TG levels (MD: −0.59 mmol/L, 95% CI: −1.21 to 0.03). Meta-regression analysis did not demonstrate any significant linear relationship between dosage of biotin supplementation and changes in TC (Coefficient = −0.03) (Supplementary Figure S1).

### Effects of biotin on HDL-C, LDL-C, VLDL-C, and TG/HDL-C levels

**Figure 6:**
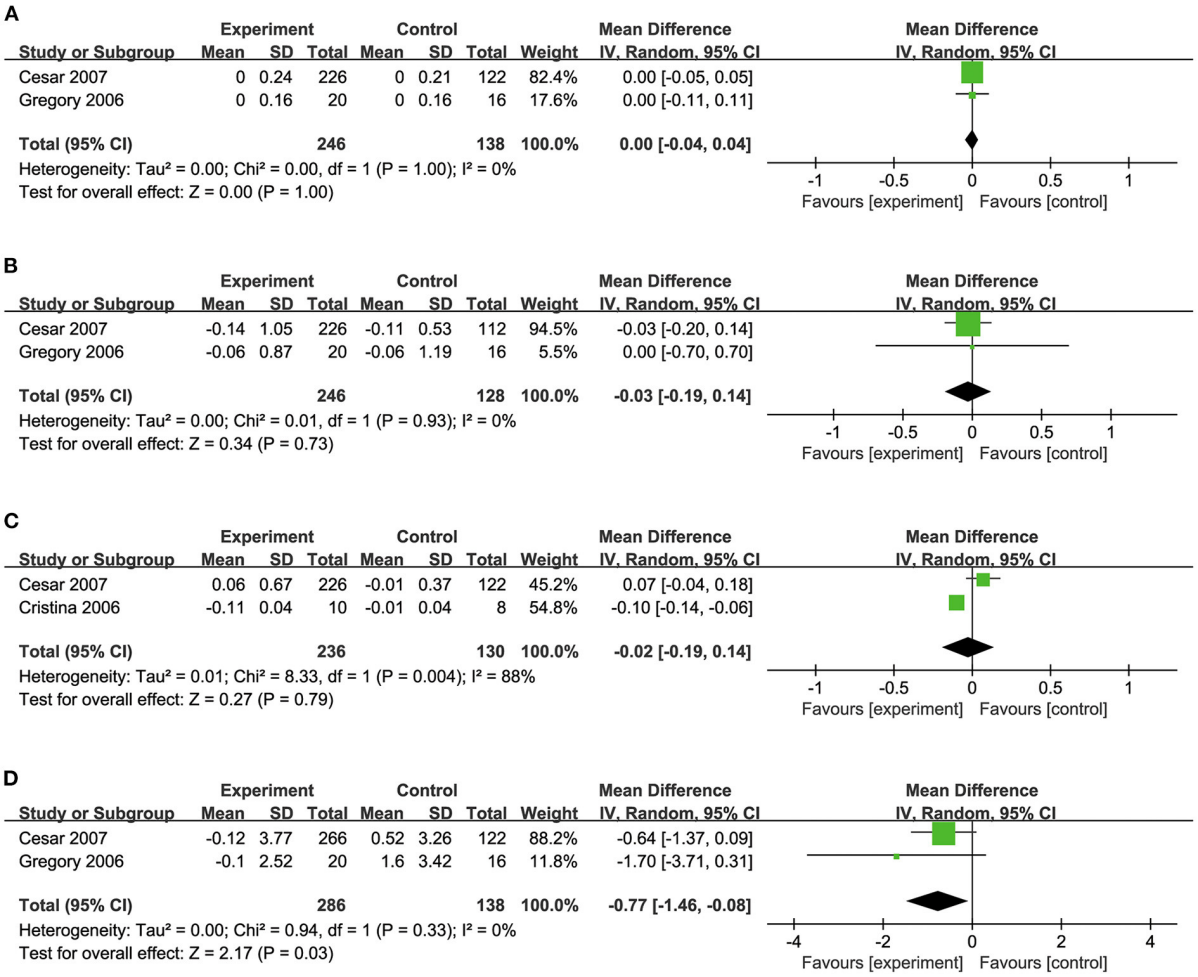
**(A)** Effects of biotin supplementation on HDL-C. **(B)** Effects of biotin supplementation on LDL-C. **(C)** Effects of biotin supplementation on VLDL-C. **(D)** Effects of biotin supplementation on TGHDL-C ratio.

Two studies (34, 39) pooled for this meta-analysis revealed no significant effect of biotin supplementation on HDL-C levels (MD: 0.00 mmol/L, 95% CI: −0.04 to 0.04) (Figure 6A). But the supplementation mildly reduced LDL-C levels (MD: −0.03 mmol/L, 95% CI: −0.19 to 0.14) and a significant decrease was found regarding TG/HDL-C ratio (MD: −0.77, 95% CI: −1.46 to −0.08) (Figures 6B,D).

Two studies (32, 34) investigated the effects of biotin supplementation on VLDL-C levels. However, no significant difference was observed in this meta-analysis (MD: −0.02 mmol/L, 95% CI: −0.19 to 0.14) (Figure 6C).

### Sensitivity analysis

**Figure 7:**
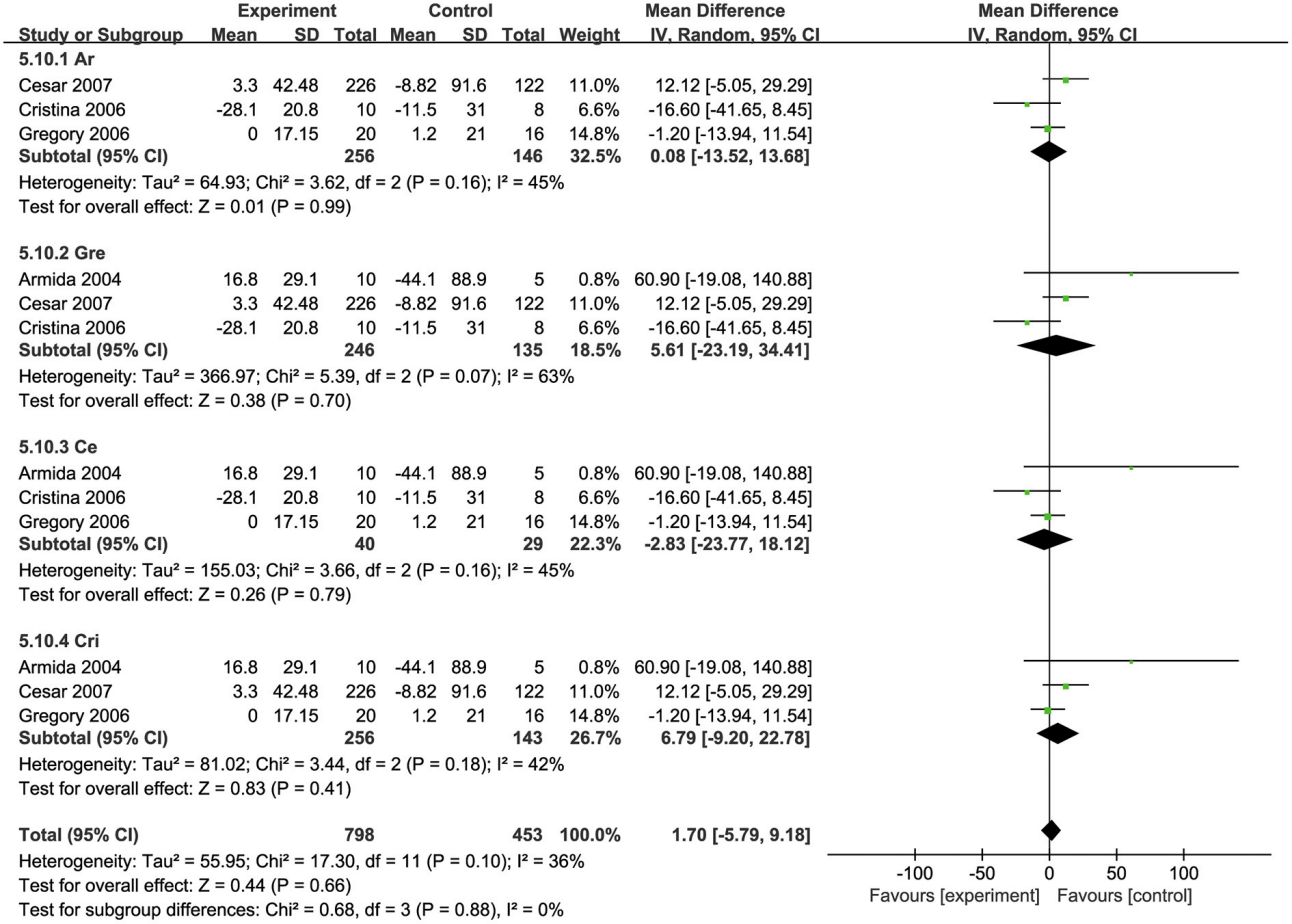
Sensitivity analysis for the effects of biotin supplementation on insulin levels.

A sensitivity analysis was also performed to test the influence of every individual trial on the overall effect size. Each trial was removed from the sensitivity analysis orderly. After removing the trails conducted by George et al. (39) or Cristina et al. (32), sensitivity analysis showed that the effects of biotin supplementation on serum insulin levels presented a significant increase [(MD: 5.61 pmol/L, 95% Cl: −23.19. to 34.41) (MD: 6.79 pmol/L, 95% Cl: −9.20 to 22.78)] (Figure 7).

## Discussion

In this meta-analysis, we evaluated the effects of biotin supplementation on glycemic control in T2DM patients. The results indicate that biotin supplementation significantly improved lipid profile by decreasing TC, TG and TG/HDL-C ratio, but no linear association was observed for these changes and biotin dosage. The influence of biotin on LDL-C, HDL-C and VLDL-C was mild to none. Supplementation with or without chromium did not cause a difference on the influence of TC and TG changes. Regarding on FBG levels, T2DM patients receiving biotin supplementation at the dosage of ≥ 9 mg/d showed a significant decrease, but the reduction was not significant on those receiving <9 mg/d according to the subgroup analysis. Moreover, results for the effect on insulin levels were inconsistent between subgroups divided by dosage of the supplement. Evidence for HbA1c was not enough because only one trial reported this parameter. Therefore, considering the small number of included studies, no conclusion can be made on the effects of biotin supplementation on insulin, HbA1c, LDL-C, HDL-C and TG/HDL-C ratio so far.

The effects of biotin on glycemic outcomes are mainly caused by two pathways. First, biotin can increase the activity and expression of glucokinase expressed in hepatocyte and pancreatic β cell (40). Glucokinase phosphorylates glucose to glucose 6-phosphate inside the hepatocyte, ensuring an adequate flow of glucose enters the cell to be metabolized (41). Glucokinase activity is essential for glucose-induced insulin secretion, post-prandial hepatic glucose uptake, and suppression of hepatic glucose output and gluconeogenesis by elevated plasma glucose (33). Diabetic patients often have subnormal hepatic glucokinase activities, which affects the rate at which glucose is converted into glucose-6-phosphate in the liver, thus hindering the breakdown of glucose and hepatic glycogen. Biotin can stimulate glucokinase to accelerate the conversion of glucose into pyruvate, thereby reducing FBG levels (41, 42). Meanwhile, after the increase of glucokinase, the carbon from glucose are provided for *de novo* lipogenesis (43). Glucokinase mRNA expression has been proven to be associated with markers of *de novo* lipogenesis and liver triglyceride content in humans (44). The overexpression of glucokinase can promote pathways that convert glucose to fatty acids, which suggests that increased glucokinase activity may leads to reduced blood glucose and induces hypertriglyceridemia and hepatic steatosis (45, 46). Secondly, biotin acts as a key coenzyme for PC in gluconeogenesis. In biotin-deficient patients, biotin administration can increase PCC activity and maintain blood glucose stability (47). Biotin can also repress both the gluconeogenic genes and their transcription factors, such as phosphoenolpyruvate carboxykinase (PCK1), glucose-6-phosphatase (G6PC), forkhead box protein O1 (FoxO1) and hepatocyte nuclear factor 4α (HNF4α) through a pathway independent of insulin-signaling (48). The role of biotin in glucokinase and PC together make up for the efficacy of its potential clinical value.

Regarding on lipid profile, biotin can regulate lipid metabolism by reducing the expression of adipogenesis genes in liver and adipose tissues. As the coenzyme of ACC1 and ACC2, biotin helps to catalyze the reaction of acetyl CoA to malonyl CoA in the synthesis of long-chain fatty acids, which is related to the synthesis of acetylcholine and cholesterol (49). Biotin supplementation can reduce lipogenesis by increasing cGMP content and activating AMP-activated protein kinase (AMPK) (50), thereby inactivating ACC1. It is also able to increase fatty acid oxidation by decreasing ACC2 activity (32). In patients with atherosclerosis and hyperlipidemia, decreased blood cholesterol concentration was found after biotin supplementation, especially those with exacerbated hyperlipidemia (51). The triglyceride-lowering effect of biotin was also reported in patients with T2DM (32). Meanwhile, another study suggested that biotin could also potentiate the suppression of appetite by upregulating ACC2 gene expression in the hypothalamus, which lead to the suppression of food intake and contribute to the prevention of diabetes (49).

Although non-human experiments have provided strong evidence for the possible effects of biotin in the prevention and treatment of diabetes, there is a huge blank regarding data from clinical trials or RCTs. The latest article included in this meta-analysis was conducted fifteen years ago. Similarly, RCTs for thiamine supplementation in T2DM subjects also dated back to nearly ten years ago (52). The notion of using vitamin a preventive or therapeutic agent for T2DM is not new, but many have focused on fat-soluble vitamins (4, 8, 9, 53). The effects of water-soluble vitamins, especially individual vitamins in the vitamin B family, have far less been emphasized on. Only a few studies have suggested the effects of vitamins C and individual vitamins within the vitamin B family in blood lipids and blood glucose levels. Results from a meta-analysis of 15 studies indicated that vitamin C supplementation significantly decreased TG and TC, but failed to improve LDL-C and HDL-C in T2DM patients (54). Another meta-analysis including six RCTs showed no significant beneficial effects of thiamine supplementation were observed on glycemic control (55).

Potential differences among included trials may be caused by the duration of diabetes, underlying health condition, dietary interference, and individual genetic and microbiota responses to biotin (56). More importantly, water-soluble vitamins could be largely expelled from the body due to T2DM patients' poor absorption ability and frequent urination. Thiamine deficiency (57), Vitamin B12 deficiency (58), niacin deficiency (59), biotin deficiency (27) and other vitamin B deficiency were widely reported in patients with T2DM. Whether and how much of the water-soluble vitamin supplementation would be lost in urine and how that influenced the ultimate effects needs further clarification. The baseline biotin level of the participants was not reported, the supplementation methods were not specified, and the dosage of biotin supplementation used in some studies was low and could easily be influenced by normal diet. All of these factors may be related to the lack of significant difference in insulin levels. Therefore, besides high quality RCTs on the effect of individual vitamin supplement, cross-sectional studies on the blood and urine vitamin levels of T2DM patients are all expected.

Limitations of this study include the quantity of included RCTs, as few trials have been conducted in this area. Besides, none of the RCTs in this meta-analysis had a treatment or follow-up period longer than three months, which could weaken the observations of long-term effect. Moreover, the dietary intake of biotin was not considered in all of the studies. Although the intervention effect did not meet the requirements for clinical significance due to the small sample sizes, we believe that our study provides important information to the current knowledge of the impact of biotin on glycemic control and lipid profile of T2DM patients.

## Conclusions

In conclusion, our systematic review and meta-analysis suggests that biotin supplementation may decrease TC and TG levels while limited evidence suggested that its influence on insulin, LDL-C, HDL-C, VLDL-C is not significant. T2DM patients receiving higher dose of biotin demonstrated a decrease in FBG levels. Biotin supplementation could be economical and potentially beneficial to T2DM patients. However, more robust-designed and updated studies with long-term follow-up and large sample sizes are expected to further evaluate the veracity of biotin supplement in T2DM patients.

## Data availability statement

The original contributions presented in the study are included in the article/Supplementary material, further inquiries can be directed to the corresponding author.

## Author contributions

YZ designed the protocol, collected, analyzed the data, and wrote the manuscript. YD collected, analyzed the data, and wrote the manuscript. YF collected the data and wrote the manuscript. YX collected and analyzed the data. YL and LZ collected the data and edited the manuscript. LW conceived the idea and edited the manuscript. All authors contributed to the article and approved the submitted version.

## Funding

This research was supported by the Faculty Research Grants of Macau University of Science and Technology (No. FRG-21-036-FMD).

## Conflict of interest

The authors declare that the research was conducted in the absence of any commercial or financial relationships that could be construed as a potential conflict of interest.

## Publisher's note

All claims expressed in this article are solely those of the authors and do not necessarily represent those of their affiliated organizations, or those of the publisher, the editors and the reviewers. Any product that may be evaluated in this article, or claim that may be made by its manufacturer, is not guaranteed or endorsed by the publisher.

## References

[1] Arokiasamy P Salvi S Selvamani Y. Global burden of diabetes mellitus: prevalence, pattern, and trends. Handbook Global Health. (2021) 21:495–538. 10.1007/978-3-030-45009-0_2832175717

[2] Cole JB Florez JC. Genetics of diabetes mellitus and diabetes complications. Nat Rev Nephrol. (2020) 16:377–90. 10.1038/s41581-020-0278-5 32398868 PMC9639302

[3] Xiong K Wang J Ma A. Adjunctive vitamin a and d for the glycaemic control in patients with concurrent type 2 diabetes and tuberculosis: a randomised controlled trial. Br J Nutr. (2022) 127:556–62. 10.1017/S0007114521001185 33820572

[4] Zhang Y Tan H Tang J Li J Chong W Hai Y . Effects of vitamin D supplementation on prevention of type 2 diabetes in patients with prediabetes: a systematic review and meta-analysis. Diabetes Care. (2020) 43:1650–8. 10.2337/dc19-1708 33534730

[5] Yu Y Tian L Xiao Y Huang G Zhang M. Effect of Vitamin D Supplementation on some inflammatory biomarkers in type 2 diabetes mellitus subjects: a systematic review and meta-analysis of randomized controlled trials. Ann Nutr Metab. (2018) 73:62–73. 10.1159/000490358 29945132

[6] Wang S Cai B Han X Gao Y Zhang X Wang R . Vitamin d supplementation for nonalcoholic fatty liver disease in type 2 diabetes mellitus: A protocol for a systematic review and meta-analysis. Medicine (Baltimore). (2020) 99:e20148. 10.1097/MD.0000000000020148 32384501 PMC7220265

[7] Barbarawi M Zayed Y Barbarawi O Bala A Alabdouh A Gakhal I . Effect of Vitamin D Supplementation on the Incidence of Diabetes Mellitus. J Clin Endocrinol Metab. (2020) 105:dgaa335. 10.1210/clinem/dgaa335 32491181

[8] Xu R Zhang S Tao A Chen G Zhang M. Influence of Vitamin E Supplementation on glycaemic control: a meta-analysis of randomised controlled trials. PLoS ONE. (2014) 9:e95008. 10.1371/journal.pone.0095008 24740143 PMC3989270

[9] Shahdadian F Mohammadi H Rouhani MH. Effect of vitamin K supplementation on glycemic control: a systematic review and meta-analysis of clinical trials. Horm Metab Res. (2018) 50:227–35. 10.1055/s-0044-100616 29523009

[10] Sahebi R Rezayi M Emadzadeh M Salehi M Tayefi M Parizadeh SM . The effects of vitamin D supplementation on indices of glycemic control in iranian diabetics: a systematic review and meta-analysis complement. Ther Clin Pract. (2019) 34:294–304. 10.1016/j.ctcp.2018.12.009 30712741

[11] Alexander-Kaufman K Harper C. Transketolase: observations in alcohol-related brain damage research. Int J Biochem Cell Biol. (2009) 41:717–20. 10.1016/j.biocel.2008.04.005 18490188

[12] Cinici E Dilekmen N Senol O Arpali E Cinici O Tanas S. Blood thiamine pyrophosphate concentration and its correlation with the stage of diabetic retinopathy. Int Ophthalmol. (2020) 40:3279–84. 10.1007/s10792-020-01513-2 32715366

[13] Valdés-Ramos R Guadarrama-López AL Martínez-Carrillo BE Benítez-Arciniega AD. Vitamins and type 2 diabetes mellitus. Endocr Metab Immune Disord Drug Targets. (2015) 15:54–63. 10.2174/1871530315666150416130242 25388747 PMC4435229

[14] Khalaf KM Khudhair MS Ashor AW. Vitamin B12 status and peripheral neuropathy in patients with type 2 diabetes mellitus. J Pak Med Assoc. (2019) 69(Suppl. 3):S40–4. 31603875

[15] Fernandez-Mejia C. Pharmacological effects of biotin. J Nutr Biochem. (2005) 16:424–7. 10.1016/j.jnutbio.2005.03.018 15992683

[16] Tong L. Structure and function of biotin-dependent carboxylases. Cell Mol Life Sci. (2013) 70:863–91. 10.1007/s00018-012-1096-0 22869039 PMC3508090

[17] Dakshinamurti K Cheah-Tan C. Biotin-mediated synthesis of hepatic glucokinase in the rat. Arch Biochem Biophys. (1968) 127:17–21. 10.1016/0003-9861(68)90195-1 5681418

[18] Romero-Navarro G Cabrera-Valladares G German MS Matschinsky FM Velazquez A Wang J . Biotin regulation of pancreatic glucokinase and insulin in primary cultured rat islets and in biotin-deficient rats. Endocrinology. (1999) 140:4595–600. 10.1210/endo.140.10.7084 10499515

[19] Sone H Ito M Shimizu M Sasaki Y Komai M Furukawa Y. Characteristics of the biotin enhancement of glucose-induced insulin release in pancreatic islets of the rat. Biosci Biotechnol Biochem. (2000) 64:550–4. 10.1271/bbb.64.550 10803952

[20] Sone H Ito M Sugiyama K Ohneda M Maebashi M Furukawa Y. Biotin enhances glucose-stimulated insulin secretion in the isolated perfused pancreas of the rat. J Nutr Biochemistry. (1999) 10:237–43. 10.1016/S0955-2863(99)00003-0 15539296

[21] De la Vega LA Stockert RJ. Regulation of the insulin and asialoglycoprotein receptors via cgmp-dependent protein kinase. Am J Physiol-Cell Physiol. (2000) 279:C2037–42. 10.1152/ajpcell.2000.279.6.C2037 11078721

[22] Lazo de la Vega-Monroy ML Larrieta E German MS Baez-Saldana A Fernandez-Mejia C. Effects of biotin supplementation in the diet on insulin secretion, islet gene expression, glucose homeostasis and beta-cell proportion. J Nutr Biochem. (2013) 24:169–77. 10.1016/j.jnutbio.2012.03.020 22841397

[23] Larrieta E Vega-Monroy ML Vital P Aguilera A German MS Hafidi ME . Effects of biotin deficiency on pancreatic islet morphology, insulin sensitivity and glucose homeostasis. J Nutr Biochem. (2012) 23:392–9. 10.1016/j.jnutbio.2011.01.003 21596550

[24] Reddi A DeAngelis B Frank O Lasker N Baker H. Biotin supplementation improves glucose and insulin tolerances in genetically diabetic Kk mice. Life Sci. (1988) 42:1323–30. 10.1016/0024-3205(88)90226-3 3280936

[25] Dakshinamurti K Modi VV Mistry SP. Some aspects of carbohydrates metabolism in biotin-deficient rats. In: Proceedings of the Society for Experimental Biology and Medicine. New York, NY: Society for Experimental Biology and Medicine (1968) 127:396–400. 10.3181/00379727-127-32699 5650996

[26] Chauhan J Dakshinamurti K. Transcriptional regulation of the glucokinase gene by biotin in starved rats. J Biol Chem. (1991) 266:10035–8. 10.1016/S0021-9258(18)99181-7 2037560

[27] Maebashi M Makino Y Furukawa Y Ohinata K Kimura S Sato T. Therapeutic evaluation of the effect of biotin on hyperglycemia in patients with non-insulin dependent diabetes mellitus. J Clin Biochem Nutr. (1993) 14:211–8. 10.3164/jcbn.14.211

[28] Coggeshall JC Heggers JP Robson Mc Baker H. Biotin status and plasma glucose in diabeticsa. Annal New York Academy Sci. (1985) 447:389–92. 10.1111/j.1749-6632.1985.tb18454.x14749229

[29] McCarty MF. In type 1 diabetics, high-dose biotin may compensate for low hepatic insulin exposure, promoting a more normal expression of glycolytic and gluconeogenic enyzymes and thereby aiding glycemic control. Med Hypotheses. (2016) 95:45–8. 10.1016/j.mehy.2016.08.002 27692165

[30] Hemmati M Babaei H Abdolsalehei M. Survey of the effect of biotin on glycemic control and plasma lipid concentrations in type 1 diabetic patients in Kermanshah in Iran (2008-2009). Oman Med J. (2013) 28:195–8. 10.5001/omj.2013.53 23772286 PMC3679599

[31] Ferre T Pujol A Riu E Bosch F Valera A. Correction of diabetic alterations by glucokinase. Proc Natl Acad Sci U S A. (1996) 93:7225–30. 10.1073/pnas.93.14.7225 8692973 PMC38964

[32] Revilla-Monsalve C Zendejas-Ruiz I Islas-Andrade S Báez-Saldaña A Palomino-Garibay MA Hernández-Quiróz PM . Biotin supplementation reduces plasma triacylglycerol and vldl In type 2 diabetic patients and in nondiabetic subjects with hypertriglyceridemia. Biomed Pharmacother. (2006) 60:182–5. 10.1016/j.biopha.2006.03.005 16677798

[33] Albarracin CA Fuqua BC Evans JL Goldfine ID. Chromium picolinate and biotin combination improves glucose metabolism in treated, uncontrolled overweight to obese patients with type 2 diabetes. Diabetes Metab Res Rev. (2008) 24:41–51. 10.1002/dmrr.755 17506119

[34] Albarracin C Fuqua B Geohas J Juturu V Finch MR Komorowski JR. Combination of chromium and biotin improves coronary risk factors in hypercholesterolemic type 2 diabetes mellitus: a placebo-controlled, double-blind randomized clinical trial. J CardioMetab Syndrome. (2007) 2:91–7. 10.1111/j.1559-4564.2007.06366.x 17684468

[35] Báez-Saldana A Zendejas-Ruiz I Revilla-Monsalve C Islas-Andrade S Cárdenas A Rojas-Ochoa A . Effects of biotin on pyruvate carboxylase, acetyl-coa carboxylase, propionyl-coa carboxylase, and markers for glucose and lipid homeostasis in type 2 diabetic patients and nondiabetic subjects. Am J Clin Nutr. (2004) 79:238–43. 10.1093/ajcn/79.2.238 14749229

[36] Moher D Liberati A Tetzlaff J Altman DG. Group^*^ P. Preferred reporting items for systematic reviews and meta-analyses: the Prisma Statement. Annal Int Med. (2009) 151:264–9. 10.7326/0003-4819-151-4-200908180-00135 21603045 PMC3090117

[37] Higgins JP Altman DG Gøtzsche PC Jüni P Moher D Oxman AD . The cochrane collaboration's tool for assessing risk of bias in randomised trials. BMJ. (2011) 343:d5928. 10.1136/bmj.d5928 22008217 PMC3196245

[38] Higgins JP Thompson SG Deeks JJ Altman DG. Measuring inconsistency in meta-analyses. BMJ. (2003) 327:557–60. 10.1136/bmj.327.7414.557 12958120 PMC192859

[39] Singer GM Geohas J. The effect of chromium picolinate and biotin supplementation on glycemic control in poorly controlled patients with type 2 diabetes mellitus: a placebo-controlled, double-blinded, randomized trial. Diabetes Technol Ther. (2006) 8:636–43. 10.1089/dia.2006.8.636 17109595

[40] McCarty MF. High-dose biotin, an inducer of glucokinase expression, may synergize with chromium picolinate to enable a definitive nutritional therapy for type Ii diabetes. Med Hypotheses. (1999) 52:401–6. 10.1054/mehy.1997.0682 10416947

[41] Adeva-Andany María M Pérez-Felpete N Fernández-Fernández C Donapetry-García C Pazos-García C. Liver glucose metabolism in humans. Biosci Rep. (2016) 36:160385. 10.1042/BSR20160385 27707936 PMC5293555

[42] Matschinsky FM Wilson DF. The central role of glucokinase in glucose homeostasis: a perspective 50 years after demonstrating the presence of the enzyme in islets of langerhans. Front Physiol. (2019) 10:148. 10.3389/fphys.2019.00148 30949058 PMC6435959

[43] Dentin R Girard J Postic C. Carbohydrate responsive element binding protein (Chrebp) and sterol regulatory element binding protein-1c (Srebp-1c): two key regulators of glucose metabolism and lipid synthesis in liver. Biochimie. (2005) 87:81–6. 10.1016/j.biochi.2004.11.008 15733741

[44] Peter A Stefan N Cegan A Walenta M Wagner S Königsrainer A . Hepatic glucokinase expression is associated with lipogenesis and fatty liver in humans. J Clin Endocrinol Metab. (2011) 96:E1126–E30. 10.1210/jc.2010-2017 21490074

[45] Morral N Edenberg HJ Witting SR Altomonte J Chu T Brown M. Effects of glucose metabolism on the regulation of genes of fatty acid synthesis and triglyceride secretion in the liver. J Lipid Res. (2007) 48:1499–510. 10.1194/jlr.M700090-JLR200 17449907

[46] Niswender KD Shiota M Postic C Cherrington AD Magnuson MA. Effects of increased glucokinase gene copy number on glucose homeostasis and hepatic glucose metabolism. J Biol Chem. (1997) 272:22570–5. 10.1074/jbc.272.36.22570 9278411

[47] Wolf B Rosenberg LE. Stimulation of propionyl coa and beta-methylcrotonyl coa carboxylase activities in human leukocytes and cultured fibroblasts by biotin. Pediatr Res. (1979) 13:1275–9. 10.1203/00006450-197911000-00014 514693

[48] Matschinsky FM. Glucokinase, glucose homeostasis, and diabetes mellitus. Curr Diab Rep. (2005) 5:171–6. 10.1007/s11892-005-0005-4 15929862

[49] Sone H Kamiyama S Higuchi M Fujino K Kubo S Miyazawa M . Biotin augments acetyl coa carboxylase 2 gene expression in the hypothalamus, leading to the suppression of food intake in mice. Biochem Biophys Res Commun. (2016) 476:134–9. 10.1016/j.bbrc.2016.04.152 27181349

[50] Aguilera-Méndez A Fernández-Mejía C. The hypotriglyceridemic effect of biotin supplementation involves increased levels of cgmp and ampk activation. BioFactors. (2012) 38:387–94. 10.1002/biof.1034 22806917

[51] Marshall M Kliman P Washington V Mackin J Weinland B. Effects of Biotin on lipids and other constituents of plasma of healthy men and women. Artery. (1980) 7:330–51. 7011260

[52] Alaei Shahmiri F Soares MJ Zhao Y Sherriff J. High-dose thiamine supplementation improves glucose tolerance in hyperglycemic individuals: a randomized, double-blind cross-over trial. Eur J Nutr. (2013) 52:1821–4. 10.1007/s00394-013-0534-6 23715873

[53] Iqbal S Naseem I. Role of vitamin a in type 2 diabetes mellitus biology: effects of intervention therapy in a deficient state. Nutrition. (2015) 31:901–7. 10.1016/j.nut.2014.12.014 26001806

[54] Namkhah Z Ashtary-Larky D Naeini F Clark CCT Asbaghi O. Does vitamin C supplementation exert profitable effects on serum lipid profile in patients with type 2 diabetes? a systematic review and dose-response meta-analysis. Pharmacol Res. (2021) 169:105665. 10.1016/j.phrs.2021.105665 33984490

[55] Muley A Fernandez R Green H Muley P. Effect of thiamine supplementation on glycaemic outcomes in adults with type 2 diabetes: a systematic review and meta-analysis. BMJ Open. (2022) 12:e059834. 10.1136/bmjopen-2021-059834 36008064 PMC9422810

[56] Voland L Le Roy T Debédat J Clément K. gut microbiota and vitamin status in persons with obesity: a key interplay. Obesity Rev. (2022) 23:e13377. 10.1111/obr.13377 34767276

[57] Page GL Laight D Cummings MH. Thiamine deficiency in diabetes mellitus and the impact of thiamine replacement on glucose metabolism and vascular disease. Int J Clin Pract. (2011) 65:684–90. 10.1111/j.1742-1241.2011.02680.x 21564442

[58] Aroda VR Edelstein SL Goldberg RB Knowler WC Marcovina SM Orchard TJ . Long-term metformin use and vitamin B12 deficiency in the diabetes prevention program outcomes study. J Clin Endocrinol Metab. (2016) 101:1754–61. 10.1210/jc.2015-3754 26900641 PMC4880159

[59] Zillikens MC van Meurs JB Sijbrands EJ Rivadeneira F Dehghan A van Leeuwen JP . Sirt1 genetic variation and mortality in type 2 diabetes: interaction with smoking and dietary niacin. Free Radic Biol Med. (2009) 46:836–41. 10.1016/j.freeradbiomed.2008.12.022 19167483

