# Supplementary material for: Influence of biotin intervention on glycemic control and lipid profile in patients with type 2 diabetes mellitus: A systematic review and meta-analysis
Source: Front Nutr. 2022 Oct 31;9:1046800. doi: 10.3389/fnut.2022.1046800 (PMC9659605; doi:10.3389/fnut.2022.1046800)
Supplement: Supplementary file 1 [file Data_Sheet_1.docx]

Supplementary Material

# Search strategy

("biotin"[MeSH Terms] OR "vitamin B7"[MeSH Terms] OR biotin"[All fields] OR "vitamin B7"[All fields] OR "vitamin b"[MeSH Terms] OR "vitamin b family"[MeSH Terms] OR "vitamin b"[MeSH Terms] OR "vitamin b complex"[MeSH Terms] OR "water soluble vitamin"[All fields] **AND** "supplementation"[Title/Abstract] OR "treatment"[Title/Abstract] OR "intervention” [Title/Abstract] OR "intake"[Title/Abstract]) **AND** ("diabetes mellitus"[MeSH Terms]) OR "diabetes"[MeSH Terms] OR "T2DM"[MeSH Terms] OR "type 2 diabetes mellitus"[All fields] OR "non-insulin dependent diabetes"[All fields] OR "urinary loss"[All fields] OR "urine loss"[All fields] OR "urination"[All fields] OR "HbA1c"[MeSH Terms] OR " glycated hemoglobin "[MeSH Terms] OR "insulin"[MeSH Terms] OR "serum insulin"[All fields] OR "fasting blood glucose"[All fields] OR "FBG"[All fields] OR "plasma glucose"[All fields] OR "FPG"[All fields] OR "cholesterol"[MeSH Terms] OR "TC"[MeSH Terms] OR "triglycerides"[MeSH Terms] OR "TG"[MeSH Terms] OR "low-density lipoprotein"[MeSH Terms] OR "HDL"[MeSH Terms] OR "high-density lipoprotein"[MeSH Terms] OR "LDL"[MeSH Terms] OR "glycemic outcomes"[All fields] OR "glycemic control"[All fields] OR "lipid profile"[All fields]) **AND** ("randomized" OR "trial" OR "controlled trials" OR "clinical trials" OR "cross-over" OR "parallel")


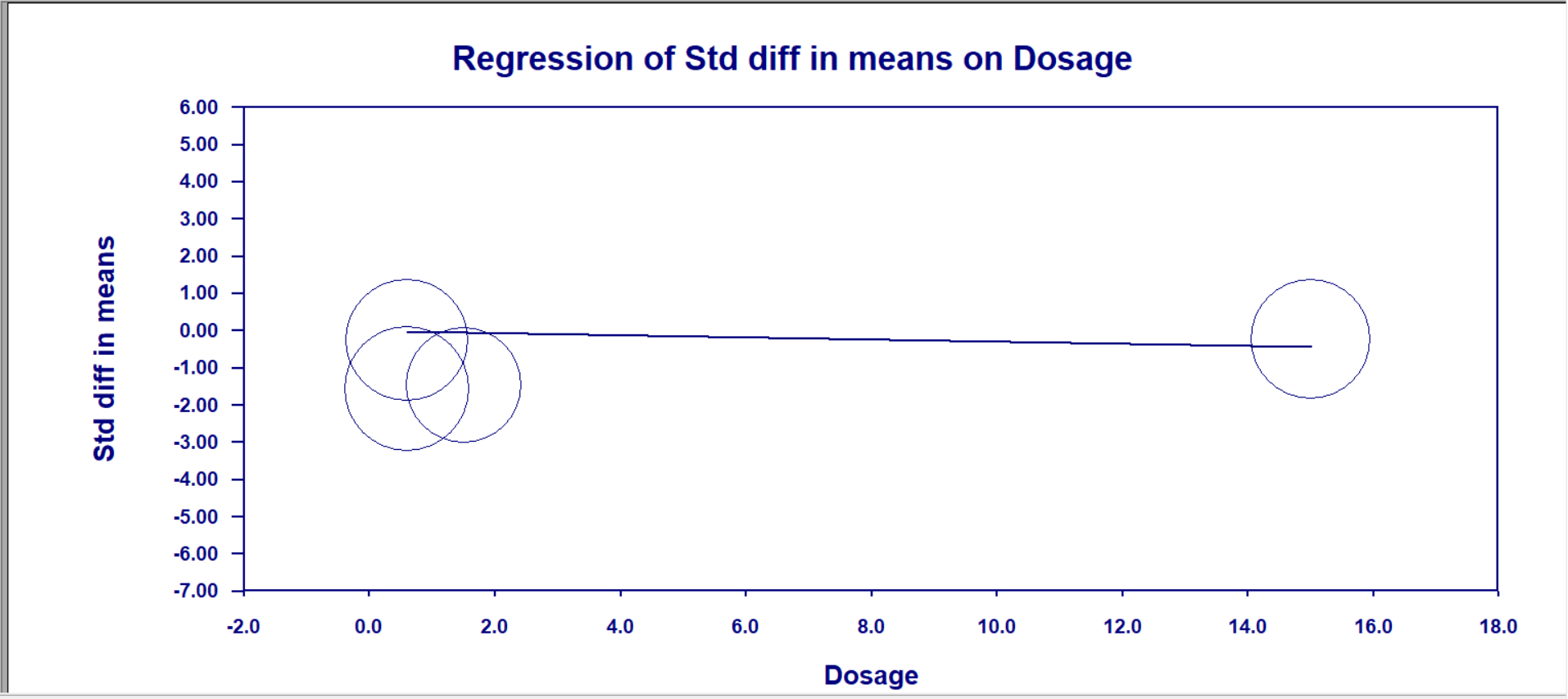


**Figure 1**. Meta-regression analysis for dosage of biotin supplementation and changes in TC


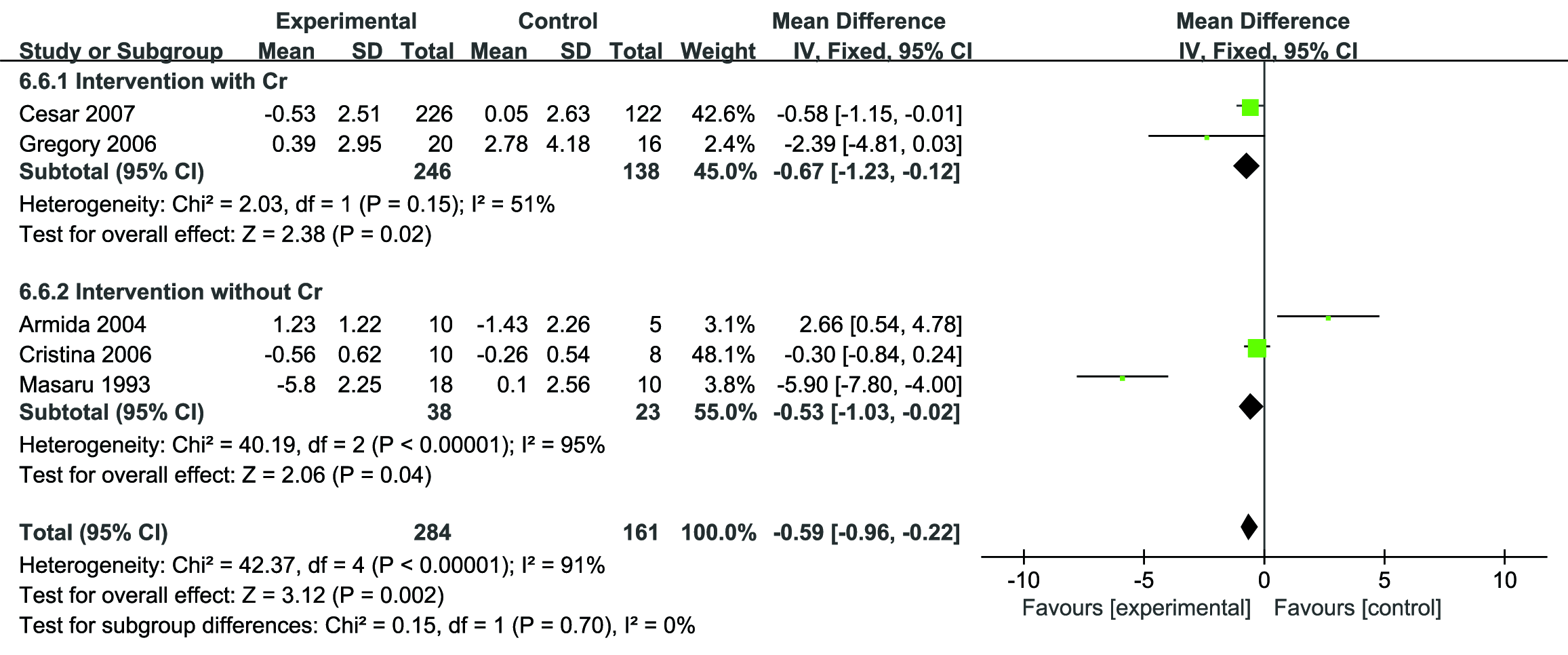


**Figure 2.** Subgroup analysis for the effects of biotin supplementation with or without Chromium on FBG


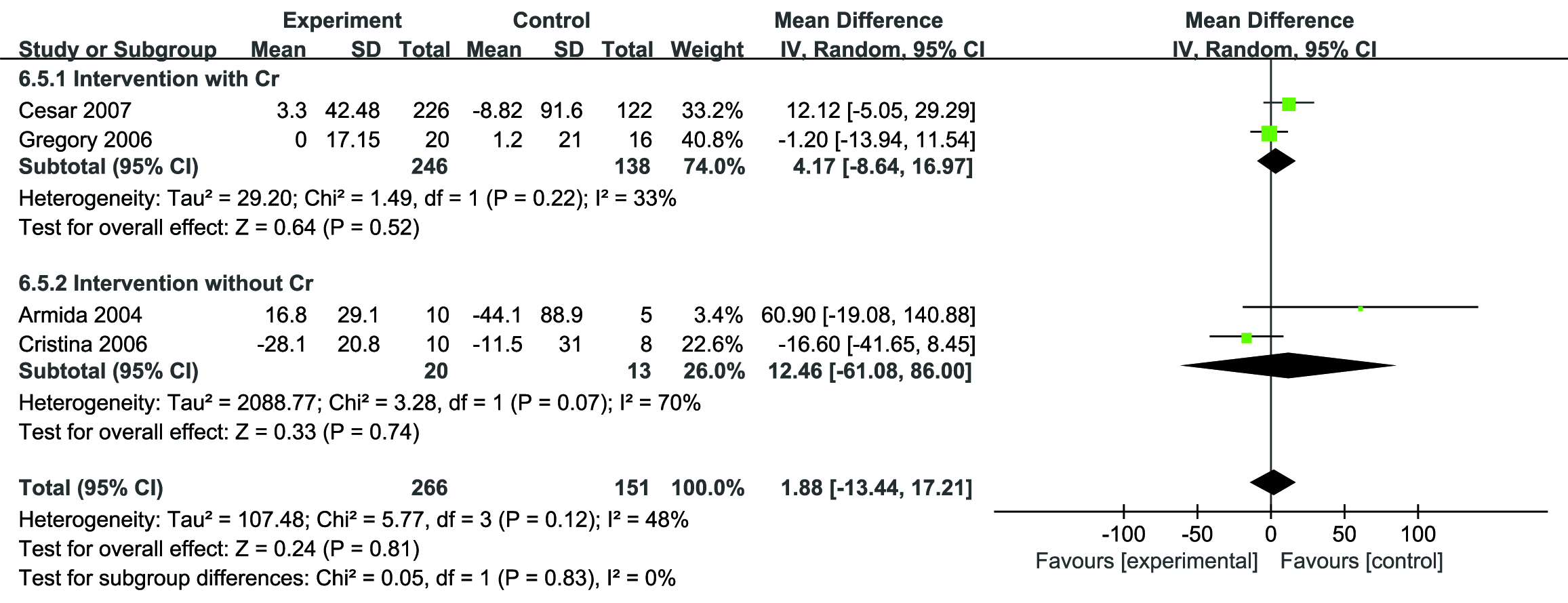


**Figure 3**. Subgroup analysis for the effects of biotin supplementation with or without Chromium on insulin
